# Elaiophylin Inhibits Tumorigenesis of Human Uveal Melanoma by Suppressing Mitophagy and Inducing Oxidative Stress *via* Modulating SIRT1/FoxO3a Signaling

**DOI:** 10.3389/fonc.2022.788496

**Published:** 2022-03-21

**Authors:** Xue Zhu, Wenjun Zou, Xinmin Meng, Jiali Ji, Xun Wang, Hong Shu, Yuan Chen, Donghui Pan, Ke Wang, Fanfan Zhou

**Affiliations:** ^1^ National Health Commission (NHC) Key Laboratory of Nuclear Medicine, Jiangsu Key Laboratory of Molecular Nuclear Medicine, Jiangsu Institute of Nuclear Medicine, Wuxi, China; ^2^ Department of Radiopharmaceuticals, School of Pharmacy, Nanjing Medical University, Nanjing, China; ^3^ Department of Ophthalmology, The Affiliated Wuxi No.2 People’s Hospital of Nanjing Medical University, Wuxi, China; ^4^ Department of Respiratory and Critical Care Medicine, The Affiliated Wuxi No.2 People’s Hospital of Nanjing Medical University, Wuxi, China; ^5^ Department of Laboratory Medicine, Cancer Medical College of Guangxi Medical University, Affiliated Tumor Hospital of Guangxi Medical University, Nanning, China; ^6^ Sydney Pharmacy School, The University of Sydney, Sydney, NSW, Australia

**Keywords:** uveal melanoma, elaiophylin, mitophagy, oxidative stress, SIRT1/FoxO3a signaling

## Abstract

Uveal melanoma (UM) is the most common primary intraocular tumor in adults, which is associated with poor prognosis. Up to 50% of UM patients develop metastasis. Therapeutics that have proven effective in cutaneous melanoma have little success in treating UM, possibly due to its low mutational burden. Therefore, new drug therapies are highly desired for UM. Our *in vitro* studies showed that Elaiophylin, a late-stage autophagy inhibitor, exhibited an outstanding anticancer activity in human UM cell lines and human UM primary cells through suppressing mitophagy, inducing oxidative stress and leading to autophagic cell death. Our mechanistic study revealed that Elaiophylin exerted its effect by down-regulating SIRT1 and thus influencing deacetylation and mitochondrial localization of FoxO3a. In our confirmatory experiments, SRT1720, a SIRT1 specific activator, could attenuate Elaiophylin-induced inhibition of mitophagy and elevation of oxidative stress, and such effects was partly reversed by FoxO3a knockdown. Our further *in vivo* studies showed that Elaiophylin dramatically inhibited tumor growth in the human UM xenograft mouse model, which was accompanied with a decreased SIRT1 expression. Thus, the current study is the first to demonstrate that Elaiophylin has a potent anti-cancer effect against UM, which activity is possibly mediated through regulating SIRT1-FoxO3a signaling axis. And Elaiophylin may be a new and promising drug candidate to treat human UM.

## Introduction

Although rare, uveal melanoma (UM) is the most common primary ocular cancer in the Caucasian population (1). Both uveal and cutaneous melanomas originate from melanocytes; however, they are significantly different regarding their pathogenesis and clinical behaviors (2). About 85% of ocular melanomas occur in the uveal tract, the vascular layer of the eye (comprising the choroid, ciliary body, and iris) (3). Approximately 50% of patients with primary UM ultimately develop distant metastasis and the median survival was reported to be less than 6 months. The liver metastasis counts up to 90% of cases (2). Overall, UM is a rare but deadly cancer. At present, the most widely used first-line treatment for this malignancy includes resection, radiation therapy and enucleation (4). Although local recurrence is extremely rare, however, it’s association with significantly higher risk of systemic metastasis highlights an urgent need of novel systematic therapies to better manage human UM.

Autophagy is an essential homeostatic and catabolic process. It sequesters misfolded proteins, damaged or aged organelles, as well as mutated proteins in double membrane vesicles called autophagosomes that ultimately fuse to lysosomes leading to the degradation of the sequestered components (5). The recycling capacity of autophagy plays a crucial role in both physiological and pathophysiological contexts and its dysregulation is associated with tumorigenesis and tumor-stroma interactions (6). Autophagy is commonly upregulated in UM and there is a strong association between extensive BECN1 overexpression and early metastases/poor prognosis (7). Autophagy inhibition may sensitize GNAQ/11-mutated UM to the MEK1/2 inhibitor, trametinib (8). Mitophagy, a specific form of autophagy, is a particular adaptation of cancer cell metabolism to recycle intracellular components in condition of metabolic stress. Its dysregulation is associated with tumorigenesis and tumor-stroma interactions (6, 9). Mitochondria are known to be the major source of intracellular reactive oxygen species (ROS) (10). The release of ROS upon mitochondrial injury induces mitophagy in order to reduce oxidative damage by eliminating impaired mitochondria and preventing ROS accumulation (11). It plays a pivotal role in reinstating cellular homeostasis in both normal and stress conditions. Increasing evidence has indicated that elevated ROS generation upon inhibiting mitophagy flux can induce lysosomal dysfunction and autophagy suppression, and finally lead to cancer cell death (12–14). Therefore, mitophagy inhibition and oxidative stress induction have been considered as new therapeutic targets in cancer treatment (15).

Elaiophylin, a C2 symmetry 16-member macrodiolide antibiotic isolated from *Streptomyces melanosporus*, is a novel late-stage autophagy inhibitor (16). Elaiophylin and its derivatives exhibit various activities including antimicrobial, anthelmintic, immunosuppressive, anti-inflammatory, antiviral and α-glucosidase inhibitory effects. Previous studies indicated that Elaiophylin exerted moderate cytotoxicity against various cancer cells such as human gastric cancer (SNU-1), hepatocellular carcinoma (SNU-354), vinblastine sensitive epidermoid carcinoma (KB-3-1) and resistant cervical cancer (KB-V1) cell lines (17). In the study of Zhao et al., Elaiophylin showed a potent antitumor efficacy as a single agent or in combination with cisplatin or under hypoxic condition in human ovarian cancer cells. Such effect of Elaiophylin was mediated through suppressing the downstream autophagosome formation (eg. inhibiting autophagosome and lysosome fusion and/or blocking the degradation of autophagic cargo inside autolysosomes) (18). Elaiophylin also exerts antitumor activity against multiple myeloma with mutant TP53 by blocking autophagy flux and subsequently inducing the persistent activation of endoplasmic reticulum stress (19). Recent studies have shown that Elaiophylin is a potent Hsp90/Cdc37 protein interface inhibitor. It interferes the interaction of Cdc37 and the N-terminus of Hsp90, and depletes Gal3, thus selectively decreases K-Ras nanoclustering (20). Hsp90/Cdc37 chaperone complex regulates mitophagy by stabilizing and activating Ulk1, which is required for the phosphorylation and release of Atg13 from Ulk1, and for the recruitment of Atg13 to damaged mitochondria (21). However, the role of Elaiophylin in UM remains unclear. SIRT1 is a member of the sirtuin family of the class III NAD^+^-dependent HDACs, which consists of seven enzymes (SIRT1 to SIRT7) that share conserved core catalytic domains but differ in their cellular localization and tissue distribution (22). Forkhead box protein O3a (FoxO3a) is one of the main effectors of cellular stress-activated signal transduction pathways. Its activity is controlled by various post-translational modifications (PTMs), which determine its subcellular localization (23). SIRT1 is known to deacetylate FoxO3a and in turn, promotes mitophagy. In the present study, we are the first to report the anti-cancer effect of Elaiophylin in *in vitro*, *ex vivo* and *in vivo* UM models, which is mediated through mitophagy inhibition, and the modulation of SIRT1-FoxO3a signaling axis.

## Methods

### Reagents

Elaiophylin was kindly provided by Prof. Xie (Institute of Medicinal Biotechnology, Chinese Academy of Medical Sciences & Peking Union Medical College, Beijing, China.) (Purity>99%), prepared as a stock solution (50 mM) in dimethyl sulfoxide (DMSO) and stored at -20°C.

### Cell Culture and Treatment

The human UM C918 and OCM1A cell lines were obtained from Beijing Beina Chuanglian Biotechnology Research Institute (BeNa Culture Collection, Beijing, China). Uveal melanoma cell line C918, derived from primary uveal melanoma and characterized by Folberg et al. (24). The human UM Mel270 cell line was purchased from BioVector NTCC Inc. (Beijing, China). The human retinal pigment epithelial cell line ARPE-19 was obtained from the National Collection of Authenticated Cell Cultures (Shanghai, China). All cells were cultured in RPMI 1640 medium with 10% (v/v) fetal bovine serum (Thermo Fisher Scientific, Waltham, MA, USA) in a humidified atmosphere of 5% CO_2_ at 37°C. Five primary UM cell lines were isolated from UM patient tumor samples and cultured with the approval from the human research ethics committee of St. Vincent’s Hospital (HREC/17/SVH/346). The primary UM cell lines were maintained in RPMI medium containing 20% FBS (v/v), 1% L-glutamine, 1% P/S, 1% ITS and 2% GCT (Thermo Scientific, Lidcombe, NSW, Australia) in a humidified atmosphere of 5% CO_2_ at 37°C.

### Cell Proliferation Assay

Cell viability was assessed by MTT assay. Briefly, cells were plated in 96-well plates at a density of 1×10^4^ cells per well. After 24 h treatment with indicated drugs, cells were incubated with 5 mg/mL MTT solution for 4 h at 37°C. The absorbance was measured at 570 nm using a SpectraMax M5 microplate reader (Molecular Devices, San Jose, CA, USA). Each experiment was performed in sextuples and repeated on three occasions. SPSS 19 was used to estimate IC_50_ values.

### Cell Apoptosis Assay

Cell apoptosis was measured with AnnexinV-FITC/PI double staining. Cells were seeded in 6-well plates at a density of 1×10^6^ cells per well. After 24 h pre-treatment with Elaiophylin (0, 0.25, 0.5 and 0.75 μM), cells were washed twice with D-PBS, and then incubated in 300 μL binding buffer (containing10 μL AnnexinV-FITC and 10 μL PI) for 15 min in the dark at room temperature. The fluorescence was analyzed by flow cytometry (Becton-Dickinson, CA, USA).

### Intracellular ROS Generation Detection

The level of intracellular ROS was determined by DCFH-DA staining. Cells were pretreated with indicated drugs for 2 h. Cells were then harvested and incubated with DCFH-DA (10 μM) for 30 min in the dark at 37°C. After staining, cells were washed twice with D-PBS. The intracellular ROS fluorescence intensity was quantified by flow cytometry and the images were taken by a fluorescence microscope (Olympus IX53; Olympus Corporation, Tokyo, Japan).

### Measurement of Mitochondrial Membrane Potential

Mitochondrial membrane potential (MMP) was measured using the lipophilic cation JC-1, which exhibits a potential-dependent amassing in mitochondria. When the mitochondrial membrane potential is high, JC-1 accumulates in the matrix of mitochondria to form polymers (J-aggregates), which can produce red fluorescence; when the mitochondrial membrane potential is low, JC-1 cannot aggregate in the matrix of mitochondria and presents as a monomer that can produce green fluorescence. Therefore, it is very convenient to detect the change of mitochondrial membrane potential through the change of fluorescence colors. The relative ratio of red to green fluorescence is often used to measure the proportion of mitochondrial depolarization. Briefly, cells (1×10^6^ cells/mL) were seeded into 6-well plates and treated with indicated drugs for 6 h. After treatment, cells were stained with JC-1 at 37°C for 30 min. The alternation of MMP level was determined by flow cytometry.

### Cell Mitochondria Isolation

The cytosol and mitochondrial fractions were isolated using Cell Mitochondria Isolation Kit (C3601, Beyotime, Shanghai, China). Cells were washed with PBS, digested with trypsin EDTA solution and collected with centrifugation (100-200g) at room temperature for 5-10 min. Then mitochondrial separation reagent was added to cells with PMSF, gently suspended the cells and place them in ice bath for 10-15 min. The cell suspension was transferred to a glass homogenizer of appropriate size and homogenize for about 10-30 times. The cell homogenates were centrifuged (600g) at 4°C for 10 min. The isolated cytoplasmic protein was precipitated with centrifugation. The supernatant was carefully transferred to another set of tubes and centrifuged (11,000g) again at 4°C for 10 min. The isolated cell mitochondria were captured in the precipitates.

### Western Blot Analysis

Cells were seeded into 6-well plates at 5×10^6^ cells per well. After treatment, cells were harvested and lysed in ice-cold RIPA buffer (Beyotime) for 30 min. Then the protein concentration was measured using BCA protein assay kit (Beyotime). A total of 50 μg protein of each sample was loaded on 15% sodium-dodecyl sulfatepoly-acrylamide gel electrophoresis and blotted onto PVDF membranes (Beyotime). The membranes were blocked with 5% non-fat milk for 1 h at room temperature and then incubated with the primary antibodies overnight at 4°C and HRP-conjugated secondary antibody for 2 h at room temperature. The protein bands were visualized using the ECL assay kit (Cat. No: P0018AM; Beyotime). The density of each band was normalized to the expression of GADPH (Cat. No: ab8245, Abcam, Cambridge, USA), VDAC1 (Cat. No: ab15895, Abcam) or Lamin A (Cat. No: ab108595, Abcam). The other primary antibodies used include Cytochrome c (Cat. No: ab13575, Abcam), SIRT1 (Cat. No: ab110304, Abcam), LC3II (Cat. No: ab192890, Abcam), PINK1 (Cat. No: ab23707, Abcam), Parkin (Cat. No: ab77924, Abcam), PGC-1α (Cat. No: ab106814, Abcam), FoxO3a (Cat. No: ab70315, Abcam), and Acetyl-K (Cat. No: ab4729, Abcam).

### Co-Immunoprecipitation Assay

Cell lysate was prepared in the immunoprecipitation buffer [1% NP-40, 150 mM NaCl, 50 mM pH7.4 Tris-HCl, 10 mM NaF, 1 mM Na_3_VO_4_, 10 mM N-ethylamide (NEM) and protease inhibitors (Complete protease inhibitor cocktail; Roche, Lewes, UK)]. For co-immunoprecipitation, 500 μg of total protein was diluted to a 500 μl total volume in lysis buffer and incubated with 10 μl of anti-Acetyl-K antibody overnight at 4°C with rocking. Immuno-complexes were captured with 30 μl of DynaBeads Protein G (Thermo Fisher Scientific, Madison, USA) for 3 h with rocking at 4°C. Protein G bead complexes were washed three times with ice-cold lysis buffer, and boiled with 1× Laemmli buffer and subjected to western blot analysis. The immunoblot was probed with anti-FoxO3a antibody (1:1,000 dilution).

### Mito-Keima Mitophagy Analysis

*Keima* gene stably expresses a natural protein that emits red and green fluorescence under acidic and central conditions respectively, which can be used for qualitative and quantitative analysis of autophagy and autophagy lysosome. The leading peptide sequence of Cox V III and *Keima* curtain form a fusion gene *mito-Keima*, and the expressed Keima protein is located in the mitochondrial matrix. When the mitochondrial autophagosome is fused with the lysosome, the fluorescence signal of Keima protein changes from green to red, which can reflect the activity of mitochondrial autophagy. Cells were transfected with the mKeima-Red-Mito-7 plasmid using Lipofectamine 3000 for 24 h and then treated with different concentrations of Elaiophylin for another 24 h. The cells were imaged using a fluorescence microscope.

### Immunofluorescent Assay

Cells growing on glass coverslips were treated accordingly, fixed with 4% PFA and blocked with 5% BSA containing 0.4% Triton X-100. Subsequently, the cells were incubated with MitoTracker Green and the primary antibodies against LAMP1 or FoxO3a at 4°C overnight and then the fluorescent secondary antibody for 1 h at room temperature. After stained with DAPI for 5 min, the cellular fluorescence was observed with a fluorescence microscope (Olympus IX53).

### Small Interfering RNA (SiRNA) Silencing

Cells were transfected with scrambled siRNA duplexes or specific siRNA duplexes targeting FoxO3a (GenePharm, Shanghai, China) using Lipofectamine 3000 according to the manufacturer’s protocol. After transfection, the cells were treated with indicated drugs and the protein expression was analysed by western blotting. The scrambled siRNA duplexes were adopted as negative controls with nontargeting sequences. The sequences of siRNAs were as following: FoxO3a siRNA (5′-AAUGUGACA-UGGAGUCCAUUA-3′); the scrambled siRNA (5′-UUCUCCGAACGUGUCA-CGUTT3′).

### Nude Mice Tumorigenesis Assay

All animals were kept in a pathogen-free environment and fed ad lib. The procedures for care and use of animals were approved by the Laboratory Animal Ethics Committee of Jiangsu Institute of Nuclear Medicine (JSINM-2020-096) and all applicable institutional and governmental regulations concerning the ethical use of animals were followed. C918 cells (3×10^7^) mixed with Matrigel at a 2:1 volume ratio was injected subcutaneously into 5-week-old BALB/c nude mice (ChangZhou Cavens Laboratory Animal Co., Ltd, Changzhou, China). When the tumor volumes reached approximately 100 mm^3^, the mice were divided randomly into two groups (n=5 per group): vehicle and Elaiophylin (2 mg/kg). The mice were then administered with vehicle or Elaiophylin by intraperitoneal injection once per day for 14 days. Body weight and tumor volumes were measured every other day. Tumor volumes were calculated as (a × b^2^)/2, where a and b were the longest and shortest diameters of the tumors, respectively. At the end of the treatment, the mice were anesthetized by intraperitoneal injection of 5 ml/kg 1% pentobarbital sodium salt. The tumors were removed, weighed, and photographed. The tumor samples were then fixed in 4% paraformaldehyde for pathological examinations.

### Preparation of [^68^Ga]Ga-NOTA-PRGD2

The fresh ^68^Ga activity was eluted from the ^68^Ge/^68^Ga generator (Isotopen Technologien München, Munich, Germany) with 0.05 M HCl at 1.5 mL per fraction into the 1.5 mL polypropylene tubes. The fraction containing the most radioactivity (~5.26 MBq) was added to 93 μL of 1M sodium acetate buffer and 50 μg of NOTA-PRGD2. The mixture was heated at 97°C for 10 min. At the end of the reaction, the activity was loaded onto a C18 column (Agilent, USA) using 10 mL deionized water and then eluted by 0.3 mL 10 mM HCl-containing ethanol.

### MicroPET Imaging and Analysis

PET scans were performed using an Inveon microPET scanner (Siemens Medical Solutions, Germany). About 3.7 MBq of 68Ga labeled tracer was administered *via* tail vein injection under isoflurane anesthesia. The dynamic image acquisitions were continued from the beginning to 60 min after the administration. For each scan, regions of interest (ROIs) were drawn using vendor software (ASI Pro 5.2.4.0) on decay-corrected whole-body coronal images. The radioactivity concentrations (accumulation) were obtained from mean pixel values within the multiple ROI volume and then converted to MBq per milliliter. These values were then divided by the administered activity to obtain (assuming a tissue density of 1 g/ml) an image-ROI-derived percent injected dose per gram (%ID/g).

### Histology and Immunohistochemistry

Tumor tissues from the vehicle and Elaiophylin groups were embedded in paraffin and cut into 8 μm-thick sections. Then, the sections were stained with H&E. For immunohistochemical staining, the sections were incubated with anti-ki67 and anti-SIRT1 antibodies overnight at 4°C and then with HRP-conjugated secondary antibodies. The sections were visualized using a DAB kit, and the images were observed using a light microscope (Olympus IX53).

### Statistical Analysis

All data were presented as the mean ± SD for a minimum of three independent experiments in triplicates. All comparisons were made using Student’s t−test or one-way ANOVA followed by Tukey’s *post hoc* test. SPSS 19.0 software package was used to analyze the data. A value of *P*<0.05 was considered as statistically significant.

## Results

### Elaiophylin Inhibits Cell Proliferation and Induces Autophagic Cell Death in C918 Cells

**Figure 1:**
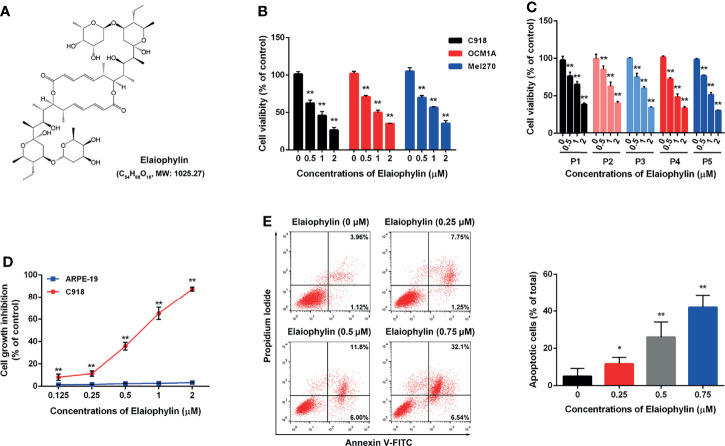
Elaiophylin inhibits cell proliferation and induces autophagic cell death in C918 cells. **(A)** The chemical structure of Elaiophylin; **(B)** C918, OCM1A and Mel270 cells were treated with different concentrations of Elaiophylin (0, 0.5, 1 and 2 μM) for 24 h. Cell viability was determined by MTT assay. **(C)** Five primary UM cells were treated with different concentrations of Elaiophylin (0, 0.5, 1 and 2 μM) for 24 h. Cell viability was determined by MTT assay. **(D)** C918 and ARPE-19 cells were treated with different concentrations of Elaiophylin (ranged from 0 to 2 μM) for 24 h. Cell viability was determined by MTT assay. **(E)** C918 cells were treated with indicated concentrations of Elaiophylin for 24h, and then apoptotic cells stained with Annexin V-FITC/PI were detected with flow cytometry. Apoptotic rates are shown in bars. Data was expressed as mean ± SD of three experiments and each experiment included triplicate repeats. ^*^ *p*<0.05, ^**^ *p*<0.01 *vs.* control.

To determine the cytotoxic effect of Elaiophylin (**Figure 1A**) in UM cells, three immortalised UM cell lines (C918 for wildtype, OCM1A for BRAF^V600E^ mutant, Mel270 for GNAQ^Q209L/P^ mutant) and five primary UM cell lines obtained from UM patient tumors were treated with different concentration of Elaiophylin for 24 h and cell viability was estimated using MTT assay. As shown in **Figures 1B, C**, Elaiophylin exhibited a dose-dependent cytotoxicity in all UM cell lines. In comparison to the human retinal pigment epithelial cell line ARPE-19 (**Figure 1D**), it inhibited C918 cell viability in a dose-dependent manner (IC_50_: 0.69 μM), with an over 80% cell death observed at 2 μM. However, ARPE-19 cells were more resistant to Elaiophylin treatment (IC_50_: 32.70 μM). Elaiophylin-incubated C918 cells were stained with Annexin V-FITC/PI and evaluated by FACS. As shown in **Figure 1E**, Elaiophylin significantly induced cell death in a concentration-dependent manner, ranged from 5.02 ± 2.43% (0 μM) to 42.04 ± 3.89% (0.75 μM). It is consistent with the report that this compound is known to be a late-stage autophagy inhibitor (18).

### Elaiophylin Induces Oxidative Stress and Mitochondrial Dysfunction in C918 Cells

**Figure 2:**
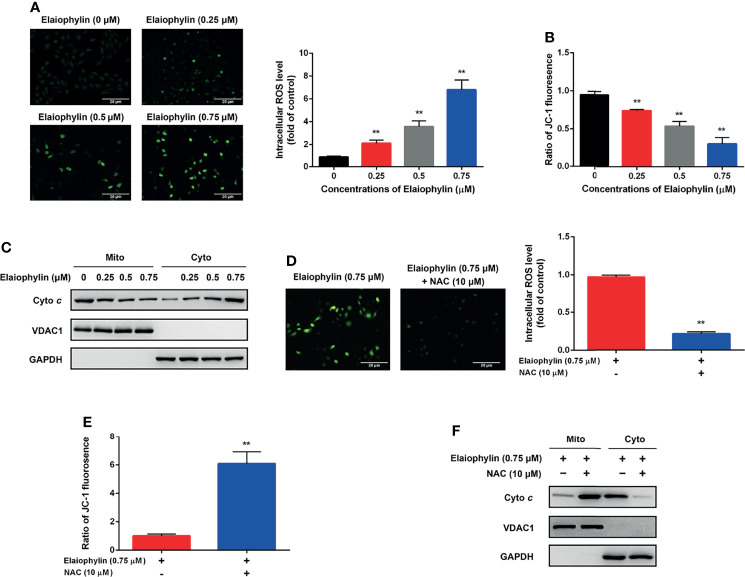
Elaiophylin induces oxidative stress and mitochondrial dysfunction in C918 cells. C918 cells were exposed to indicated concentrations of Elaiophylin, with or without NAC (10 μM) pre-treatment. **(A, D)** The representative images of ROS measurement (green fluorescence) in C918 cells after 2 h exposure to Elaiophylin. Intracellular ROS levels were detected by DCFH-DA fluorescent probe. The representative images of fluorescent probe were shown on the left and the quantitative analysis of ROS level was shown on the right. **(B, E)** The MMP of C918 cells exposed to indicated concentrations of Elaiophylin, with or without NAC. **(C, F)** Lysates of C918 cells exposed to indicated concentrations of Elaiophylin, with or without NAC were separated into cytoplasmic and mitochondrial fractions. Cytochrome *c* translocation was measured by western blotting. GAPDH and VDAC1 were used as loading controls for cytoplasm and mitochondria, respectively. Data was expressed as mean ± SD of three experiments. ^**^ *p*<0.01 *vs.* control.

Oxidative stress-mediated mitochondrial dysfunction is the inductive factor of autophagic cell death. To assess the effect of Elaiophylin on mitochondrial function, the intracellular ROS generation and MMP were evaluated. The intracellular ROS level was evaluated with DCFH-DA fluorescent probe. As shown in **Figure 2A**-ROS level (green), the results indicated that intracellular ROS level was significantly increased in a concentration-dependent manner in C918 cells upon exposure to different concentrations of Elaiophylin. As shown in **Figures 2B, C**, the MMP was significantly reduced upon the treatment of Elaiophylin, in accompany with the releasement on cytochrome *c* from the mitochondria into the cytoplasm. Pre-treatment with 10 μM NAC (N-acetylcysteine, widely used as an antioxidant) for 6 h dramatically attenuated the Elaiophylin-induced mitochondrial dysfunction in C918 cells (**Figure 2D**-ROS level (green), and **Figure 2F**). Taken together, our findings suggested that Elaiophylin-induced autophagic cell death was associated with ROS-mediated mitochondrial dysfunction in C918 cells.

### Elaiophylin Inhibits Mitophagy by Down-Regulating SIRT1 in C918 Cells

**Figure 3:**
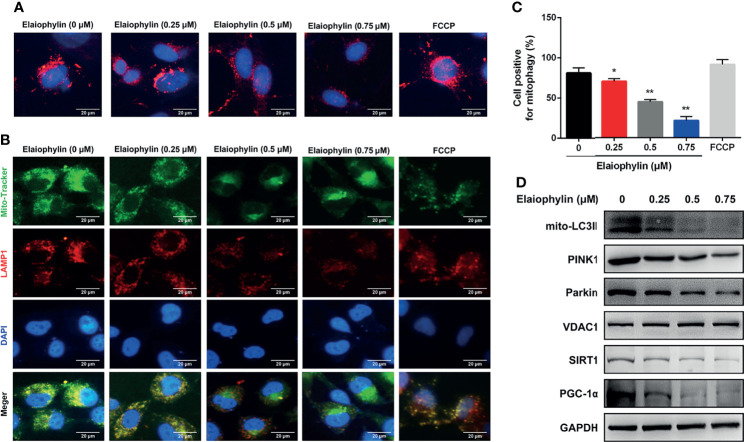
Elaiophylin inhibits mitophagy in C918 cells. **(A)** C918 cells overexpressing Mito-Keima plasmid was treated with Elaiophylin for 24 h. Mito-Keima (red fluorescence) was detected by a fluorescence microscope. FCCP, a mitophagy agonist, was used as positive control. **(B)** Colocalization of mitochondria and lysosomes. Mitochondria were stained with MitoTracker Green (200 nM), and lysosomes were stained with LAMP1 (red fluorescence). The representative images of fluorescent labeling were shown here. **(C)** Quantitative analysis of cellular mitophagy of **(B)** was conducted with Image J software. Five different visual fields of each group were selected for measurement. **(D)** Western blotting was performed to analyze the expression of proteins related to mitophagy. GAPDH and VDAC1 were used as loading controls for cytoplasm and mitochondria, respectively. Data was expressed as mean ± SD of three experiments. ^*^ *p*<0.05, ^**^ *p*<0.01 *vs.* control.

Mitophagy is an important cell protective mechanism against oxidative stress by cleaning up defective mitochondria. To explore the role of Elaiophylin in mitophagy, C918 cells were transfected with mito-Keima plasmid, a pH-sensitive fluorescent protein located in mitochondria. As shown in **Figure 3A**-Mito-Keima (red), red fluorescence was observed in untreated C918 cytoplasm, indicating the occurrence of mitophagy at physiological level. FCCP, an activator of mitophagy as well as mitochondrial oxidative phosphorylation uncoupling agent, was used as the positive control. Importantly, Elaiophylin significantly suppressed mitophagy in a concentration-dependent manner. In addition, the colocalization of mitochondria with LAMP1 (a lysosome marker shown as red fluorescence), demonstrated mitophagy inhibition by Elaiophylin (**Figure 3B**-Mito Tracker (green), LAMP1 (red), DAPI (blue) and 3C). Furthermore, compared to the control, mitochondrial autophagosome marker light chain 3 (LC3)-II (mito-LC3II) expression was downregulated in response to Elaiophylin treatment, as well as that of PINK1 and Parkin, two key factors that regulate mitophagy (**Figure 3D**).

**Figure 4:**
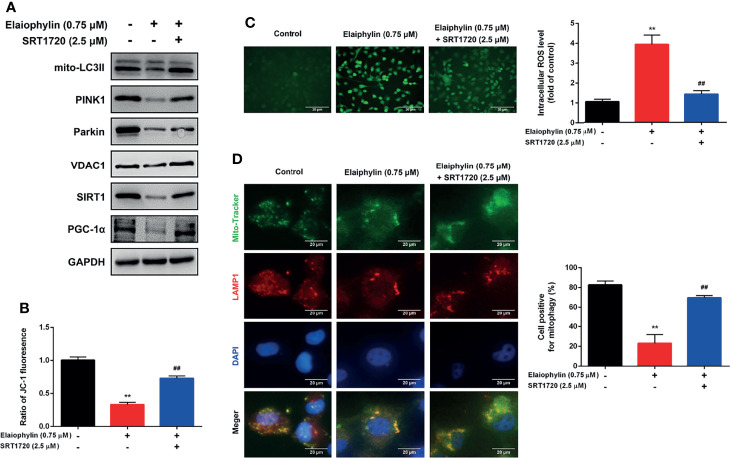
Elaiophylin inhibits mitophagy in C918 cells by regulating SIRT1. After pre-treatment with SRT1720 (a SIRT1 specific activator) for 24 h, C918 cells were incubated with Elaiophylin for another 24 h. **(A)** Western blotting was performed to analyze the proteins related to mitophagy. GAPDH and VDAC1 were used as loading controls for cytoplasm and mitochondria, respectively. **(B)** The membrane potential of C918 cells pre-treatment with or without SRT1720 followed by the incubation with or without Elaiophylin was evaluated by JC-1 fluorescent probe. **(C)** Intracellular ROS level was determined by DCFH-DA fluorescent probe. The representative images of fluorescent probe were shown on the left and the quantitative analysis of ROS level was shown on the right. **(D)** Colocalization of mitochondria and lysosomes. Mitochondria were stained with MitoTracker Green (200 nM), and lysosomes were stained with LAMP1 (red fluorescence). The representative images of fluorescent labeling were shown on the left and the quantitative analysis of cellular mitophagy was shown on the right (Image J was used for quantitative analysis of fluorescence co-localization, and five different visual fields of each group were selected for measurement). Data was expressed as mean ± SD of three experiments. ^**^ *p*<0.01 *vs.* control, ^##^ *p*<0.01 *vs.* Elaiophylin group.

SIRT1 (silent information regulator of transcription 1) is a key regulator of autophagy/mitophagy and mitochondrial function. We found that the expression of SIRT1 and its downstream target PGC-1α (proliferator-activated receptor-γ coactivator α) were both reduced in Elaiophylin-treated C918 cells (**Figure 3C**). SIRT1 activator SRT1720 reduced intracellular ROS generation and restored MMP in Elaiophylin-treated C918 cells [(**Figures 4A–C)**-ROS level (green)]. And the inhibitory effect of Elaiophylin on the fusion of mitochondria and lysosomes was significantly attenuated by SRT1720 [(**Figure 4D)**-Mito Tracker (green), LAMP1 (red), DAPI (blue)]. Moreover, SRT1720 treatment increased the expression of related proteins that downregulated by Elaiophylin (**Figure 4A**). Together, these data indicated that Elaiophylin inhibited mitophagy activity by down-regulating SIRT1 in C918 cells.

### Elaiophylin Regulates SIRT1 by Modulating FoxO3a Deacetylation and Mitochondrial Localization in C918 Cells

**Figure 5:**
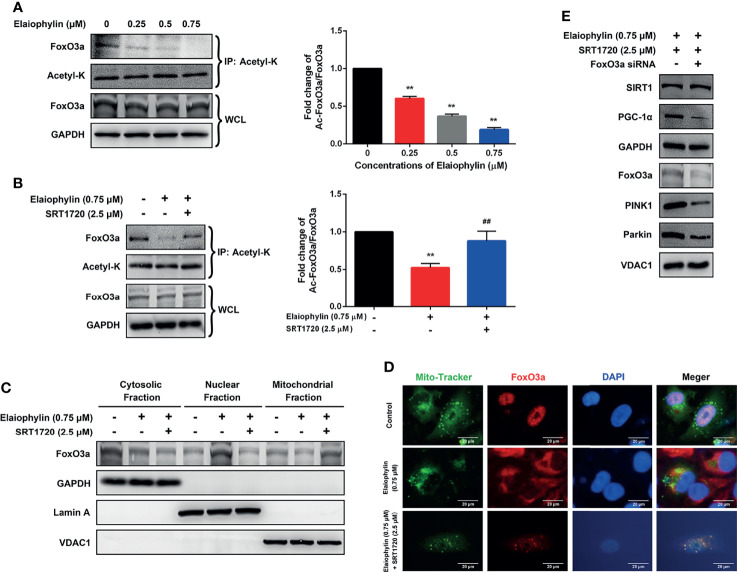
Elaiophylin modulates SIRT1 in C918 cells by manipulating FoxO3a deacetylation and mitochondrial localization. **(A, B)** After treatment with indicated drugs, cells were divided into two groups. A portion of lysate was immunoprecipitated with anti-Acetyl-K antibody and the rest was directly subjected to immunoblotting. The representative images were shown on the left and the densitometry analysis of protein expression was shown on the right. **(C)** Cell lysate fractions (ie. cytosolic, nuclear, and mitochondrial fractions) were prepared and subjected to immunoblotting analysis. GAPDH is the cytosolic marker; LaminA is applied as the nuclear marker; and VDAC1 is adopted as the mitochondrial marker. **(D)** Colocalization of mitochondria with FoxO3a by immunofluorescence **(E)** The expression of typical proteins related to mitophagy. Western blotting was performed to analyze the representative proteins in mitophagy. GAPDH and VDAC1 were used as loading controls for cytoplasm and mitochondria, respectively. Data was expressed as mean ± SD of three experiments. ^**^ *p*<0.01 *vs.* control, ^##^ *p*<0.01 *vs.* Elaiophylin group.

Elaiophylin First, whether the acetylation of the SIRT1 substrate FoxO3a, could be modulated by Elaiophylin was assessed. To investigate the level of acetylated FoxO3a, co-immunoprecipitation assay was used with Acetyl-K antibody to pull all acetylated proteins down. As shown in **Figure 5A**, Elaiophylin treatment resulted in a concentration-dependent induction of FoxO3a acetylation, while SRT1720 could potently attenuate such effect (**Figure 5B**). To identify the subcellular localization of FoxO3a, cell lysate was separated to nuclear, mitochondrial, or cytosolic fractions. As shown in **Figure 5C**, FoxO3a showed a more prominent localization in the nuclear fraction upon Elaiophylin treatment than that in the cytosol fraction. Interestingly, the activation of SIRT1 significantly increased the accumulation of FoxO3a in the mitochondrial fraction [(**Figures 5C, D)**-Mito Tracker (green), FoxO3a (red), DAPI (blue)]. Our findings showed that Elaiophylin may facilitate FoxO3a leakage into the nucleus, which was dramatically attenuated by SIRT1 activation.

To confirm the role of FoxO3a in SIRT1-mediated mitophagy, FoxO3a knockdown was exerted by siRNA in C918 cells. As shown in **Figure 5E**, FoxO3a knockdown significantly attenuated the SIRT1 activation-enhanced mitophagy-related protein expression, in particular PGC-1α, PINK1 and Parkin, in Elaiophylin-treated C918 cells. The findings indicated that SITR1-mediated FoxO3a deacetylation and mitochondrial translocation play critical roles in the inhibitory effect of Elaiophylin on mitophagy in C918 cells.

### Elaiophylin Suppresses Tumor Growth in a C918-Xenograft Model by Inhibiting SIRT1-Mediated Mitophagy

**Figure 6:**
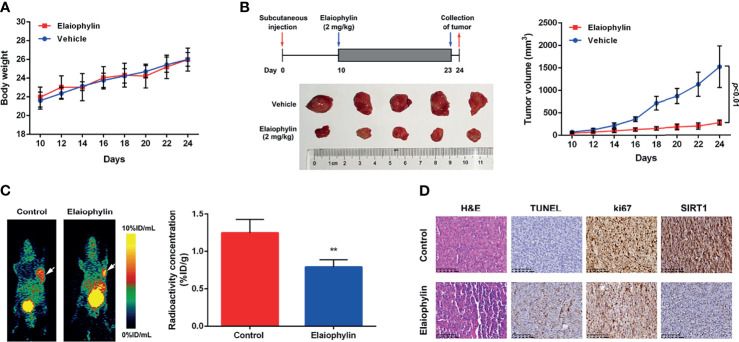
Elaiophylin suppresses tumor growth in the C918-xenograft model by inhibiting SIRT1-mediated mitophagy. **(A)** Body weight of xenograft mice were measured every other day. **(B)** Tumor volumes were measured every other day. At the end of the treatment, the tumors were removed and photographed (n=5). The images of tumors were shown on the left and the tumor growth curve was displayed on the right. **(C)** *In vivo* PET imaging of C918-xenografted mice injected with [^68^Ga]Ga-NOTA-PRGD_2_. Tumors are indicated by arrows. ROIs are shown as mean %ID/g ± SD. **(D)** H&E staining of tumor sections of C918-xenograft mice. TUNEL and immunohistochemistry staining of ki67 and SIRT1 were performed. The representative images of staining are shown here. Data was expressed as mean ± SD of three experiments. ^**^ *p*<0.01 *vs.* control.

To determine the *in vivo* anti-UM effect of Elaiophylin, C918 cells were xenografted into immunodeficient nude mice. The xenograft mice received intraperitoneal injections of either vehicle or Elaiophylin (2 mg/kg) every day for 14 days. No significant difference in the mean body weight was observed between the control and Elaiophylin treatment group (**Figure 6A**). Elaiophylin at the current dose was well tolerated in mice with no toxic events encountered throughout the course of treatment, such as agitation, impaired movement and posture, indigestion, or diarrhea. As shown in **Figure 6B**, tumor volume was significantly reduced in Elaiophylin-treated group compared to that of the control. Dynamic PET scanning was also performed after one hour post injection of the radiotracer [^68^Ga]Ga-NOTA-PRGD_2_. **Figure 6C**-white arrow showed representative PET images of mice bearing C918 xenograft tumor with or without Elaiophylin treatment. The control group displayed markedly higher uptake of radiotracer in the tumor than the Elaiophylin-treated group. Hematoxylin and eosin (H&E) staining of tumor sections of xenograft mice Elaiophylin demonstrated morphological changes in the treatment group, as indicated by signs of necrosis with infiltration of inflammatory cells and fibrosis (**Figure 6D**). Elaiophylin treatment dramatically increased the numbers of TUNEL-positive cells, which is indicative of apoptosis in the treatment group compared to that of the control group. Additionally, Elaiophylin treatment inhibited the expression of Ki67 (**Figure 6D**). In addition, Elaiophylin treatment significantly decreased the level of SIRT1 in the tumors of xenograft mice. All these findings indicated that Elaiophylin suppressed xenograft UM tumor growth by inhibiting SIRT1-mediated mitophagy.

## Discussion

Mitophagy-induced mitochondrial clearance is an important cancer survival mechanism in response to oxidative stress and mitochondrial injury. New agents that can inhibit this process have attracted a lot of attentions in cancer drug development. In this study, we reported for the first time that Elaiophylin, a late-stage autophagy inhibitor, potently inhibits UM cell proliferation by inhibiting mitophagy. Elaiophylin-induced ROS generation and mitochondrial dysfunction cannot be reversed by low-level mitophagy. Additionally, our mechanistic study revealed that SIRT1-medicated FoxO3a deacetylation and mitochondrial translocation play critical roles in the anti-UM effect of Elaiophylin.

Sirtuins are a family of mammalian class III histone deacetylases with a fundamental role in sensing and modulating cellular response to external stress and therefore involved in aging, oxidative stress control, inflammation, differentiation, and cancer development (25). Recent studies indicated that sirtuins’ activation contributes to the control of autophagy and mitophagy. During transcriptional and post translational modifications of proteins related to the autophagy and mitophagy machinery, sirtuins control ROS production or the major metabolic pathways in cancer cells (26). SIRT1 is the most prominent and extensively studied member of sirtuins. As a primary nuclear protein, SIRT1 greatly influences mitochondrial biogenesis and turnover (26). SIRT1-regulated macro-autophagy has been widely considered as a cellular protective mechanism against stress and death insults (22, 27). Accumulating evidence demonstrated that SIRT1 is involved in oxidative stress and might promote mitophagosome formation through deacetylating key autophagy-related molecules in the form of NAD^+^-dependence (28, 29). PINK1 (PTEN induced putative kinase 1) and the E3 ubiquitin ligase Parkin are two critical factors involved in SIRT1-mediated mitophagy in response to oxidative stress (30–32). ROS generation and mitochondrial membrane potential dissipation stabilize PINK1 on the outer mitochondrial membrane (OMM), facilitate the phosphorylation and E3 ligase activity of Parkin, and induce the phosphorylation of ubiquitin molecules at Ser65 on OMM proteins (33, 34). These proteins further recruit autophagy receptors (eg. NBR1, p62, NDP52, OPTN and TAX1BP1) to mitochondria and subsequently engulf into the autophagosomes for degradation (14). In the present study, SIRT1 is down-regulated by Elaiophylin treatment, which in turn, decreases the level of PGC-1α and autophagosome marker light chain 3 (LC3)-II as well as inhibits PINK1/Parkin accumulation in the mitochondria. SIRT1 activator SRT1720 partly attenuates the above effects that influenced by Elaiophylin.

Besides its nuclear localization, the majority of SIRT1’s known functions are associated with deacetylation of transcriptional factors, such as FOXO, tumor suppressor p53, and nuclear factor-kappa B (NF-κB) (35, 36). FoxO3a is a member of the forkhead box (FOX) family (class O subfamily), and is a core regulator of cellular homeostasis, stress response and longevity, since it can modulate a variety of stress responses upon nutrient shortage, oxidative stress, hypoxia, heat shock and DNA damage (37, 38). The precise regulation of FoxO3a is likely enacted by an intricate combination of post-translational modifications (PTMs), such as phosphorylation, acetylation, methylation, and ubiquitination, which determine its subcellular localization (23, 39). In particular, FoxO3a PTMs drive FOXO3a towards the nuclear and/or mitochondrial compartment in response to stress stimuli. FoxO3a regulates a set of specific genes involved in the modulation of various cellular processes. In fact, cytoplasmic FoxO3a is inactive and shuttled either to the nucleus or the mitochondria to exert its transcriptional function. Over accumulation of ROS can cause oxidative damage to mitochondria, leading to mitophagy through an SIRT1-dependent mechanism (40). Activated SIRT1 modulates FoxO3a transcriptional activity by specific deacetylation. Deacetylated FoxO3a translocates to mitochondria and inhibits mitochondrial activity by lowering the mtDNA copy number, reducing the expression of mitochondrial genes and directly interacting with PGC-1α (41, 42). PGC-1α regulates mitochondrial biogenesis but also impacts on mitochondrial quality control machinery including fission, fusion, and mitophagy (43). Sirt1-deacetylated FoxO3a also induces mitophagy by promoting the expression of Bnip3, an atypical BH3-only protein known as a pro-apoptotic factor, which thus protects cells from mitochondrial dysfunction (44). In this study, we observe that Elaiophylin treatment in C918 cells increased FoxO3a acetylation and cytoplasm localization. And the SIRT1 activator SRT1720 potently attenuated such effect by inducing FoxO3a deacetylation and translocation to the mitochondria. FoxO3a knockdown significantly inhibited SIRT1-mediated mitophagy.

Other members of sirtuins have also been linked to the control of autophagy and mitophagy by modulating transcription of autophagy and mitophagy genes, by post translational modification of proteins related to the autophagy and mitophagy machinery, by controlling ROS production or major metabolic pathways such as Krebs cycle or glutamine metabolism. SIRT2 interacts and deacetylates FoxO1 and suppresses its induction of autophagic cell death by un-interacting with ATG7 (45). Downregulation of SIRT2 also increases basal autophagy in colorectal cancer cells protecting them from mitotic catastrophe caused by microtubule inhibitors (46). SIRT3 controlling mitophagy represents an important mechanism to prevent mitochondrial dysfunction and apoptosis in tumor cells under hypoxia. SIRT3 silencing causes a decrease of mitochondriogenesis and an increase of mitochondrial dysfunction and ROS leading to an activation of autophagy and apoptosis. SIRT3 overexpression has been linked to poor survival in many cancer patients, including ER positive breast cancer and colorectal cancer (47). SIRT4 also plays an important role in mitochondrial morphology/quality control and regulation of mitophagy. Moderate overexpression of SIRT4 accompanied by increased levels of the inner-membrane bound long form of the GTPase OPA1 (L-OPA1) promotes mitochondrial fusion, and sensitized cells to mitochondrial stress (48). SIRT5 controls ammonia detoxification by regulating CPS1 in liver mitochondria. Ammonia-induced autophagy and mitophagy are regulated by SIRT5. It upregulates autophagy markers MAP1LC3B, GABARAP, and GABARAPL2, mitophagy markers BNIP3 and the PINK1-PARK2 system (49). SIRT6 has pleiotropic protective actions, including anti-inflammatory and anti-apoptotic effects, and promotes autophagy in podocytes (50). SIRT7-mediated autophagic response plays a protective role against cell death and the inhibition of SIRT7 has a potential to improve the efficacy of anti-metabolic therapy in non-small cell lung cancer cells (51). The role of each sirtuin member in cancer cells forms the basis of their potential application as adjuvant anti-cancer therapies. Natural and synthetic activators or inhibitors of sirtuins have been developed and these molecules seem to hold promising results against cancers. Further study on the regulatory mechanism of Elaiophylin on sirtuins may lead to new targets to treat relevant tumors.

UM is a rare but deadly cancer. The treatment of the primary tumor has made significant improvement with the introduction of globe-preserving approaches; however, metastatic disease remains a critical issue due to the lack of effective therapeutic strategies. UM is not sensitive to traditional chemotherapeutic drugs and immunotherapies barely have any effect. Despite lower efficacy demonstrated in initial ICI studies, there are a number of ongoing clinical trials investigating novel immunotherapy approaches in UM, including vaccine, adoptive T cells, and combination immunotherapy trials. The induction of autophagy may also benefit tumor cells escape from immune surveillance and result in intrinsic resistance against anti-tumor immunotherapy (52). Therefore, autophagy inhibitor such as Elaiophylin may play a critical role in the combination immunotherapy in UM. The acquired drug resistance of molecular targeted drugs is the primary challenge in treating UM. Cancer cells reutilize their mitochondria to facilitate cancer progression and acquire drug resistance. Oncometabolites, such as fumarate and 2-hydroxyglutarate, may promote resistance by upregulating the nuclear factor erythroid 2-related factor 2 (Nrf2) pathway, inhibiting the anti-tumor immune response, or promoting angiogenesis (53). Therefore, targeting autophagy or mitophagy may be potential and effective UM therapeutics (54). Zhou et al. identified a novel autophagy/mitophagy inhibitor liensinine sensitizes breast cancer cells to chemotherapeutic drugs through DNM1L dephosphorylation and mitochondrial translocation-mediated mitochondrial fission (12). Moreover, microRNA and long noncoding RNAs (lncRNAs) are proved to be critical regulators in numerous cellular processes, including autophagy and mitophagy. MicroRNAs overexpressed in high-risk UM, such as miR-17-5p, miR-21-5p, and miR-151a-3p, target 106 genes involved in the pathways like cell cycle regulation, EGF signaling and EIF2 signaling (55). LncRNA ZNNT1, as a major downstream effector of MTOR, promotes ATG12 transcription and subsequently induces cell death, leading to the suppression of UM tumorigenesis. Cui et al. reported a total of 13 differentially expressed autophagy genes were identified and validated in Gene Expression Omnibus, and 11 autophagy-related lncRNAs were found to be associated with overall survival. Several biological processes and signaling pathways were enriched in the high-risk group, including Toll-like receptor signaling pathway, natural killer cell-mediated cytotoxicity, and B- and T-cell receptor signaling pathway (56). Chen et al. also predicted 6 autophagy-related lncRNAs that are potential molecular biomarkers and treatment targets for UM patients, which were significantly concentrated in the biological pathways related to cytoplasmic component recycling, energy metabolism, and apoptosis (57).

**Figure 7:**
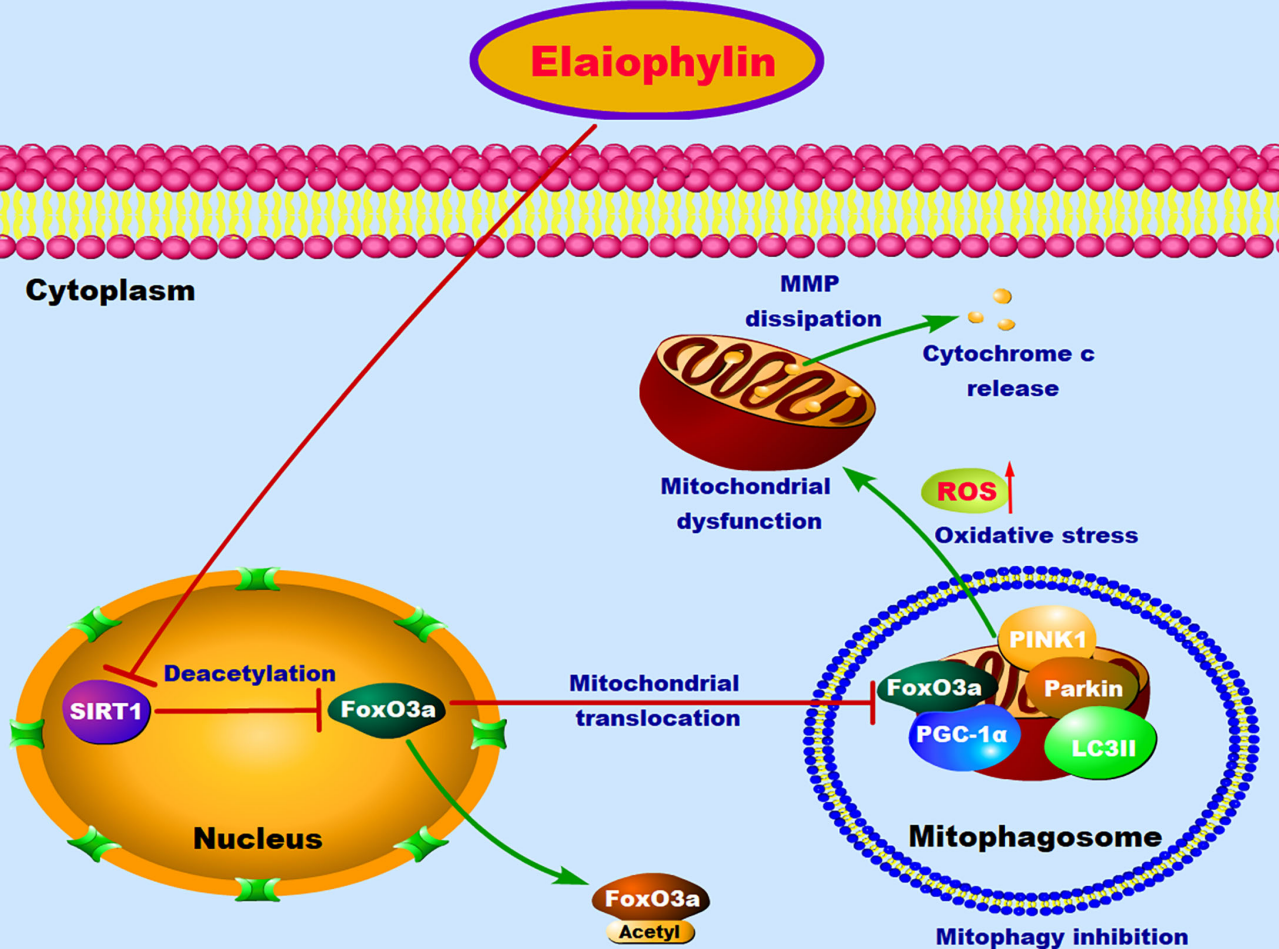
The proposed molecular mechanism of Elaiophylin in killing UM cells. In normal condition, FoxO3a deacetylated by SIRT1 results in its translocation to mitochondria, where deacetylated FoxO3a and PGC-1α form the FoxO3a/PGC-1α complex. Increased formation of FoxO3a/PGC-1α complex triggers ubiquitination and PINK1/Parkin-mediated proteasomal degradation for mitochondrial clearance. Elaiophylin treatment inhibits SIRT1-mediated mitophagy, promotes mitochondrial dysfunction and oxidative stress, which eventually leads to the death of UM cells.

In conclusion, our results suggested that Elaiophylin inhibits UM proliferation and induces apoptosis by increasing oxidative stress. SIRT1-mediated antioxidant regulation is dependent on FoxO3a deacetylation and mitochondrial localization. Elaiophylin can down-regulate the expression of SIRT1, increase FoxO3a acetylation and cytoplasm localization, as well as suppress mitophagy to clean up damaged mitochondria (**Figure 7**). These novel anti-cancer properties of Elaiophylin may have important clinical implications in developing new therapeutic agents for UM.

## Data Availability Statement

The original contributions presented in the study are included in the article/supplementary materials. Further inquiries can be directed to the corresponding authors.

## Ethics Statement

Five primary UM cell lines were isolated and cultured from UM patient tumor samples with the approval from the human research ethics committee of St. Vincent’s Hospital (HREC/17/SVH/346). The patients/participants provided their written informed consent to participate in this study. All animals were kept in a pathogen-free environment and fed ad lib. The procedures for care and use of animals were approved by the Laboratory Animal Ethics Committee of Jiangsu Institute of Nuclear Medicine (JSINM-2020-096) and all applicable institutional and governmental regulations concerning the ethical use of animals were followed.

## Author Contributions

XZ, WZ, and KW designed experiments. XZ, WZ, XM, JJ, YC, DP, and FZ carried out experiments. XZ, WZ, XW, HS, and FZ analyzed experimental results. XZ and WZ wrote the manuscript. WZ and KW finished the final version approval. All authors contributed to the article and approved the submitted version.

## Funding

This work was supported by Major project of Wuxi Commission of Health (Z202009, Z202014), Six talent peaks project in Jiangsu Province (No. WSW-047), Six-one Scientific Research Project (No. LGY2019087), Young and middle-aged top medical and health talents project of Wuxi Commission of Health (BJ2020031), Postdoctoral Science Foundation funded project of Jiangsu Province (2021K196B).

## Conflict of Interest

The authors declare that the research was conducted in the absence of any commercial or financial relationships that could be construed as a potential conflict of interest.

## Publisher’s Note

All claims expressed in this article are solely those of the authors and do not necessarily represent those of their affiliated organizations, or those of the publisher, the editors and the reviewers. Any product that may be evaluated in this article, or claim that may be made by its manufacturer, is not guaranteed or endorsed by the publisher.
